# Novel behavior in a polymer solution: the disappearance of the melting temperature (T_m_) and enthalpy change (ΔH_m_) of the solvent

**DOI:** 10.1038/s41598-020-70331-4

**Published:** 2020-08-07

**Authors:** Mi Rae Kim, Hee Jung Park, Kang Ho Cheon, Choong Kyun Yeom, Kee Yoon Lee

**Affiliations:** 1 Department of Polymer Science and Engineering, Chungnam National University, 99 Daehak-Ro, Yuseong-Gu, Deajeon, 34134 Republic of Korea; 2 Western Seoul Center, Korea Basic Science Institute, 150 Bugahyeon-Ro, Seodaemun-Gu, Seoul, 03759 Republic of Korea; 3 SepraTek, 730 Gyejok-Ro, Daedeok-Gu, Daejeon, 34396 Republic of Korea

**Keywords:** Polymer chemistry, Chemistry

## Abstract

The phase change temperature and enthalpy change as a function of polystyrene (PS) concentration in dimethylformamide through a dynamic heating and quenching process were investigated. Cold crystallization, freezing and melting phenomena in a 10 wt% PS solution were all observed. Cold crystallization and melting phenomena were still observed in a 20 wt% solution. In a 30 wt% solution, all three phenomena disappeared without any solvent enthalpy changes, e.g., enthalpy changes at the melting temperature. The disappearance of both the melting temperature and the melting enthalpy change indicated that all polymer and solvent molecules in the 30 wt% solution existed only in the amorphous phase without any phase changes despite repeated heating and quenching processes. Thus, our results can provide a new approach for gelation through enthalpy changes and can be applied in the fabrication of porous membranes with a narrow distribution.

## Introduction

Polymer chains can be completely extended in a very dilute solution when they are dissolved in an appropriate solvent. A single polymer chain is usually in a coil form in solution due to balanced interactions with the solvent and the polymer itself^1^. Molecular interactions with solvents and polymers have typical properties that can be applied to many industrial processes, and they play important roles in applications, such as membrane casting and formation^2–6^, battery separator preparation^7–9^, and polymer synthesis^10,11^, as well as in determining solubility parameters^12–15^. Recently, a 30 wt% polystyrene (PS) solution in dimethylformamide (DMF) was studied in an ultralow temperature region by Samitsu et al^16^. While preparing mesoporous polymer nanofiber networks by flash freezing the polymer solution, the authors observed a cold crystallization temperature (T_c_) at − 85 ~ − 65 °C. The change in T_c_ with polymer concentration suggested that the crystallization of the solvent in the polymer-rich phase was limited.

Additionally, the molecular interaction between polymer and solvent is closely related to the gelation of the polymer solution^17–20^ and has been studied in thermal insulation^21^, thermoreversible gels^22–24^ and size-controlled microgels^25^. Gelation can occur by physical linking or by chemical linking between the chains, resulting in a single macroscopic molecule where the gel point is defined and the solution loses fluidity and suddenly becomes very viscous^26^. The gelation of a polymer solution has been studied mainly by various gel formation mechanisms, such as the attraction between molecules as a function of temperature^27^, the formation of organometallic complexes^26^, and the attractive interaction between a few hydrophobic groups^28,29^.

The polymer and solvent that constitute the polymer solution have their own melting temperature (T_m_) and enthalpy change (ΔH_m_). When the polymer in the polymer solution is the main component, the polymer’s T_m_ is observed, the solvent’s T_m_ is not observed, and the glass transition temperature (T_g_) of the polymer varies with the polymer type and the affinity of the solvent^30^. In this study, the polymer was not considered alone, but the polymer solution in which the solvent is the main component was the focus.

We studied the phenomena of the solvent in solution in which both the enthalpy change (ΔH_c_) at the T_c_ and the enthalpy change (ΔH_m_) at the T_m_ of the solvent upon heating became zero, and thus, the T_m_ and T_c_ of the solvent disappeared. In this regard, this thermal behavior is discussed further in comparison with conventional gelation. To understand the characteristics of the solvent itself in polymer solutions, the T_c_ and T_m_ of DMF were measured through a dynamic cooling and heating scan process. The T_c_, T_m_, ΔH_c_, and ΔH_m_ of the solvent in the polymer solution consisting of PS and DMF were measured and compared. In addition, a new approach for understanding the gelation phenomena was investigated by examining T_m_ and ΔH_m_ by varying the concentration of the polymer solution.

Further studies were carried out in solutions of PS and *N*-methyl-2-pyrrolidone (NMP), polyethersulfone (PES) and DMF, and PES and NMP, as shown in the Supplementary Information. Based on the experimental results, we constructed a schematic diagram showing the molecular states between the polymer chains and solvent molecules at different polymer concentrations as a function of temperature.

## Results

### Phase change behavior of solvents

**Scheme 1:**
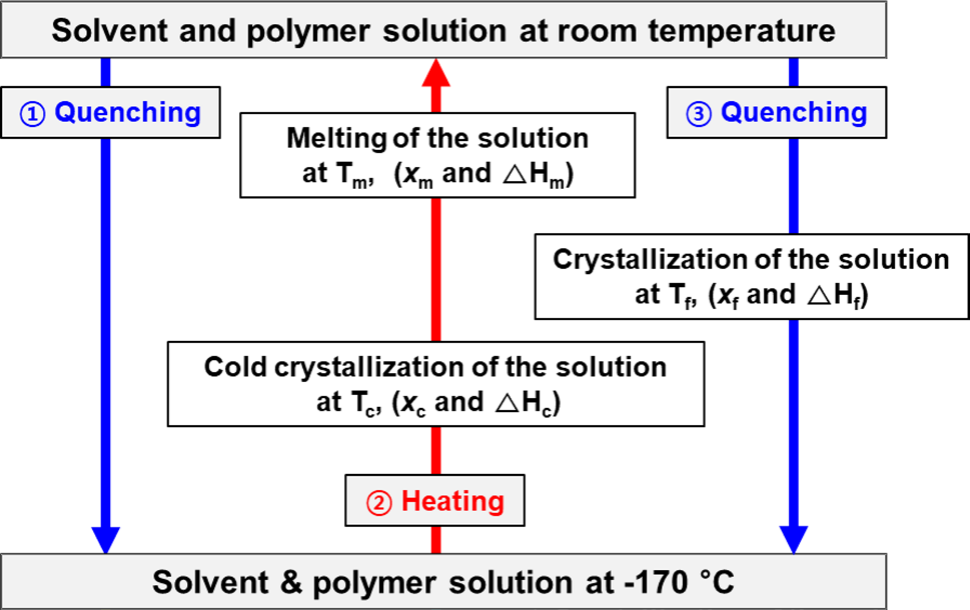
Schematic diagram of a dynamic quenching and heating scan process programmed for the DSC measurement of solvent and polymer solutions (x_c_: degree of crystallization of the solution at T_c_, x_m_: degree of crystallization changes at T_m_, x_f_: and degree of crystallization of the solution below the freezing point, T_f_).

A dynamic quenching and heating scan process by differential scanning calorimetry (DSC) is shown schematically in Scheme 1.

The DSC measurement procedure was carried out by a sequential temperature process, as shown in Scheme 1, in which the solvent or polymer solution was quenched at room temperature, the temperature was decreased to an ultralow temperature and held at − 170 °C for 5 min to stabilize the solution, and then the solution was heated to room temperature and quenched again. The supercooled solvent molecules that were not partially crystallized and existed in the frozen amorphous state became active as the temperature increased, resulting in a cold crystallization phenomenon below T_m_. Then, during the heating scan, all previously formed crystals were melted at the T_m_, and when they were cooled again, crystallization reversibly occurred at T_f_. At T_f_, T_c_, and T_m_, corresponding enthalpy changes (ΔHs) and changes in the degree of crystallization (x) occurred because phase changes took place in which the crystalline and amorphous phases reversibly changed.

**Figure 1:**
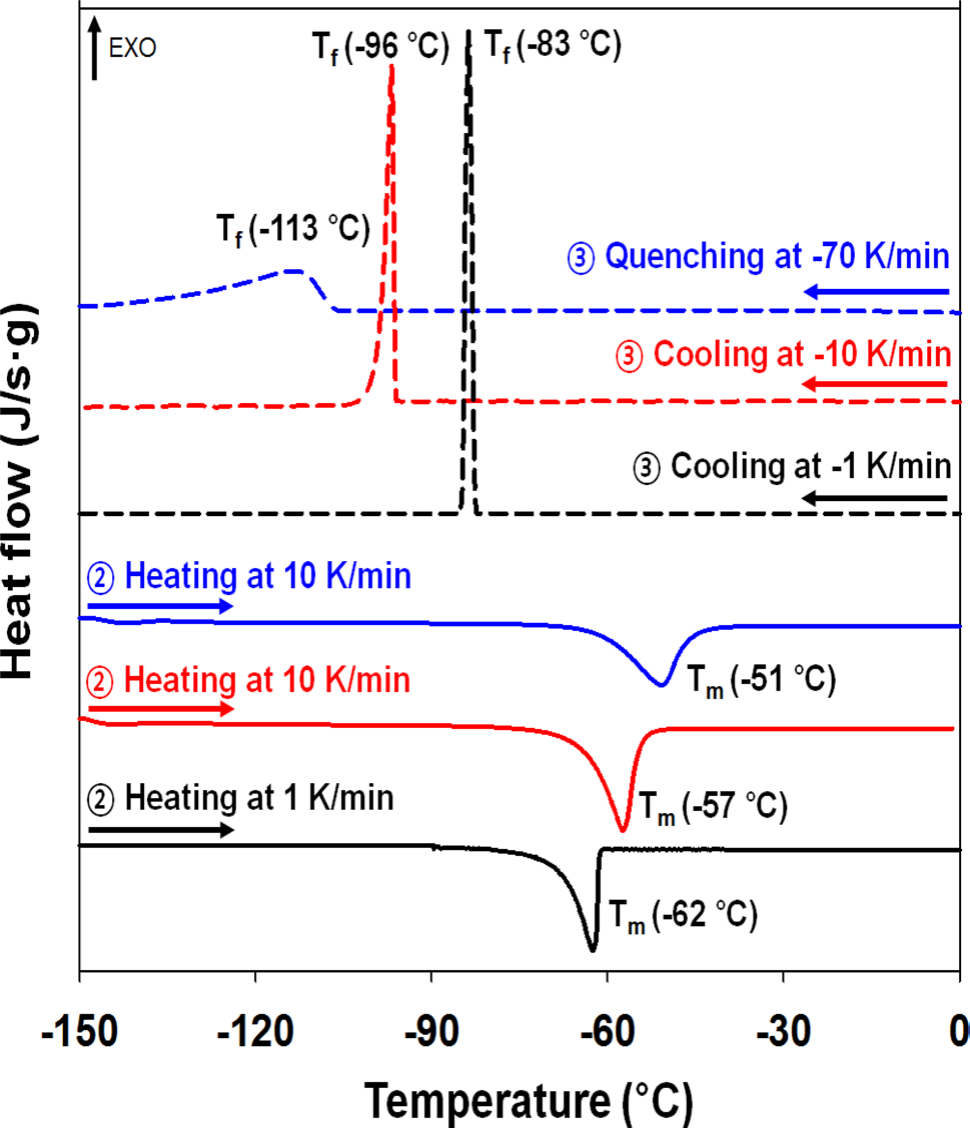
Dynamic DSC heat flow graphs in the process of cooling, heating, and cooling of DMF at room temperature. Solid line: heating, dotted line: cooling, and the same color line: one cycle (black line: heating and cooling at − 1 K/min, red line: heating and cooling at − 10 K/min, and blue line: heating at 10 K/min and quenching at − 70 K/min).

First, to observe the crystallization phenomenon of the pure solvent, T_f_ and T_m_ were measured by changing the heating and cooling rates according to Scheme 1, as shown in Fig. 1. Note that steps ① quenching and ③ quenching in Scheme 1 were performed at the same cooling rate. The DSC graph was plotted with the data measured during steps ② heating and ③ quenching shown in Scheme 1. Here, the data in ① quenching were not used to eliminate the thermal history. The method for determining T_f_ and T_m_ in the DSC thermogram is described in detail in the Methods section, where ΔHm was calculated by integrating the peak area in the DSC thermogram.

**Figure 2:**
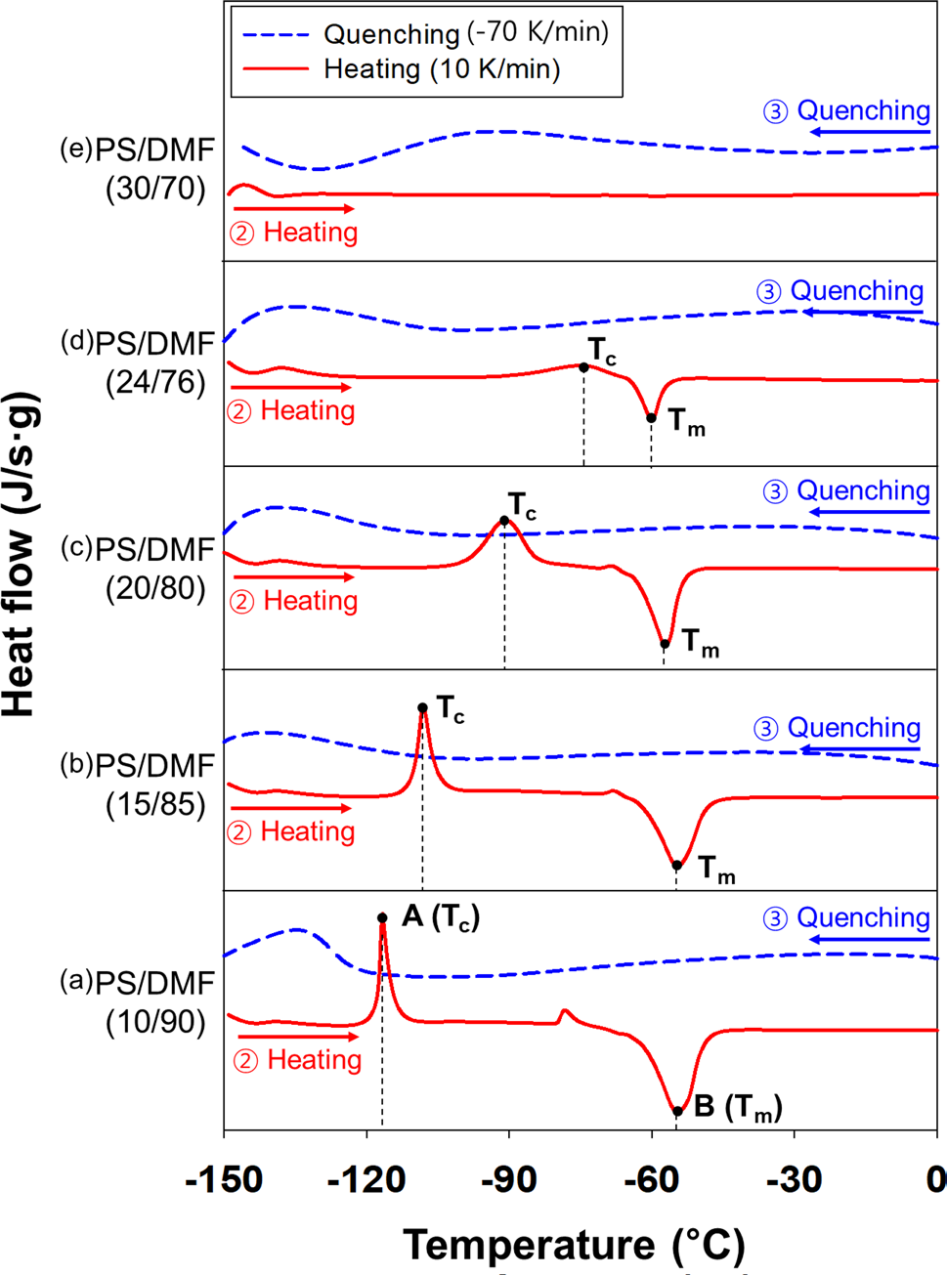
DSC graph obtained during the heating (red solid line) and quenching (blue dotted line) process for polymer solutions (PS/DMF) as a function of PS concentration and scan temperature.

Here, the DSC thermograms of Figs. 1 and 2 are plotted and were compared by shifting them vertically for a clear comparison. Cooling the DMF at room temperature with varying rates of − 70 K/min, − 10 K/min and − 1 K/min, T_f_ appeared at − 113 °C, − 96 °C and − 83 °C, respectively, and T_m_ was observed at − 60 ± 10 °C. Thus, T_f_ was greatly affected by the cooling rate, and the measured ΔH_f_ was − 94 J/g with − 1 K/min and − 121.6 J/g with − 10 K/min. The value of ΔH_m_ was determined to be 128 J/g. When the cooling rate was − 10 K/min, a small difference was observed in the value of ΔH_f_ (− 122 J/g), which is the enthalpy change in pure DMF molecules from an amorphous liquid state to a crystalline state, and ΔH_m_ (128 J/g) is assumed due to the DSC measurement error. The literature values for the T_m_ and ΔH_m_ of DMF are 212.9 K (− 60.1 °C) and 122.4 J/g, respectively^31^.

### Crystallization behavior of polymer solutions

The experimental results of the polymer solutions of PS and DMF according to Scheme 1 are shown in Fig. 2. The temperature and enthalpy change behaviors were investigated when the PS concentrations increased to 10, 15, 20, 24 and 30 wt%. The DSC graph was plotted with data measured during steps ② heating (10 K/min) and ③ quenching (− 70 K/min), as shown in Scheme 1.

When the polymer solution was supercooled to below T_f_, the solvent tended to nucleate and crystallize to reduce the Gibbs free energy^32–34^. As shown in Fig. 2a, a 10 wt% PS solution (PS/DMF (10/90)) was quenched at − 70 K/min and then heated at 10 K/min to increase the mobility of amorphous state DMF molecules, and at point A (T_c_), cold crystallization occurred, resulting in an exothermic peak (ΔH_c_). When the temperature was increased to melting point B (T_m_) of the solvent, the solvent became a liquid, and the crystalline phase of the molecules disappeared and became an amorphous liquid phase. At this time, ΔH_m_ appeared as an endothermic peak. Considering the general phase change, the following Eq. (1) can be given, since the enthalpy changes are state functions.

1 $$\Delta {\text{H}}_{{\text{c}}} + \Delta {\text{H}}_{{\text{m}}} + \Delta {\text{H}}_{{\text{f}}} = \, 0.$$

In Scheme 1, the sum of ΔH_f_ due to the phase change at T_f_ in step ③ quenching, the ΔH_c_ produced by cold crystallization in step ② heating, and the ΔH_m_ during melting in step ② heating are “0”.

Here, if we represent x_f_ and x_c_ as the degree of crystallization at T_f_ and T_c_, respectively, and if x_m_ is the degree of crystallization change at T_m_ to the amorphous phase, then x_m_ = x_f_ + x_c_, since all crystals disappear at T_m_. When the solution was quenched at all concentrations in Fig. 2, the DSC heat flow showed a distinct curve from − 110 to − 150 °C. This phenomenon shows the mechanical characteristics of the DSC process. That is, when the solution was quenched to approximately − 170 °C with a fast cooling rate of − 70 K/min, heat was applied to match this cooling rate, and the curve appeared to show an unstable baseline, which was due to equipment problems. Therefore, it was difficult to accurately measure ΔH_f_ in the ultralow temperature region below − 100 °C, and the value of ΔH_f_ was replaced by the value calculated by Eq. (1). Since there were changes in ΔH_f_ and ΔH_m_ due to the effect of the polymer on the T_f_ and T_m_ of the solution, ΔH_f,D_ and ΔH_m,D_ were introduced to represent these quantitative values. ΔH_m,D_ is the difference at T_m_ between the calculated enthalpy change and the experimentally measured enthalpy change when the solvent weight fraction excluding the polymer in the solution is considered as pure solvent and calculated. Thus, the values ΔH_m,D_ and ΔH_f,D_ can be a factor in determining the polymer influence on the pure solvent. Equations (2) and (3) show the calculation methods of ΔH_f,D_ and ΔH_m,D,_ respectively. At the T_f_ of the pure solvent, a freezing enthalpy change value of − 122 J/g was used, similar to the literature value, and at the T_m_ of the pure solvent, an experimental enthalpy change value of 128 J/g was used.

2 $$\Delta {\text{H}}_{{{\text{f}},{\text{D}}}} = { 122 }* \phi_{1} + \Delta {\text{H}}_{{\text{f}}} \left( {{\text{J}}/{\text{g}}} \right),$$

3 $$\Delta {\text{H}}_{{{\text{m}},{\text{D}}}} = { 128 }* \phi_{1} - \Delta {\text{H}}_{{\text{m}}} \left( {{\text{J}}/{\text{g}}} \right),$$

where ${\phi }_{1}$ is the weight fraction of DMF in the polymer solution. Table 1 shows the measurement values in Figs. 1 and 2.

**Table 1:** Phase change temperatures and enthalpy change values of DMF solvent and polymer solutions (PS/DMF) measured by DSC^a^.

	T_f_ (°C)	T_c_ (°C)	T_m_ (°C)	ΔH_f_ (J/g)	ΔH_f,D_^d^ (J/g)	ΔH_c_ (J/g)	ΔH_m_ (J/g)	ΔH_m,D_^e^ (J/g)
DMF (− 70 K/min)	− 113	–	− 51	− 93	–	–	120	–
DMF (− 10 K/min)	− 96	–	− 57	− 122^b^	–	–	128	–
DMF (− 1 K/min)	− 83	–	− 62	− 96	–	–	98	–
PS/DMF (10/90)	–	− 116	− 54	− 61^c^	49	− 42	103	12
PS/DMF (15/85)	–	− 108	− 54	− 39^c^	65	− 48	87	21
PS/DMF (20/80)	–	− 90	− 57	~ 0	98	− 62	61	41
PS/DMF (24/76)	–	− 75	− 60	–	–	− 18	20	77
PS/DMF (30/70)	–	–	–	–	–	0	0	90

^a^Collected from Figs. 1 and 2.

^b^The measured ΔH_f_ value was − 121.6 J/g with a scan rate of 10 K/min, and the reported literature value is − 122.4 J/g. We used − 122 J/g for ΔH_f_.

^c^ΔH_f_ was calculated by Eq. (1).

^d^ΔH_f.D_ was calculated by Eq. (2).

^e^ΔH_m.D_ was calculated by Eq. (3).

In Fig. 2a, the PS/DMF (10/90) solution showed the T_c_ and T_m_ at − 116 °C and − 54 °C, respectively. Considering the T_f_ values of the pure DMF solvent to be where T_c_ was not seen (Fig. 1 and Table 1) and considering the ΔH_c_ value of − 42 J/g of the PS/DMF (10/90) solution, some pure solvent molecules formed crystallites, and the other molecules remained in the amorphous frozen phase, resulting in cold crystallization during the heating scan. For the PS/DMF (10/90) solution in Table 1, ΔH_f,D_ was 49 J/g and ΔH_m,D_ was 12 J/g because the number of DMF crystallites in the solution produced with PS interference was less than that produced in the pure DMF solution. That is, the hindered crystallization seemed to be due to steric hindrance as well as the molecular interaction between the polymer and solvent^35^. The experimental measurements were reproducible, as shown in Supplementary Fig. 9. In Fig. 2b, when the PS concentration was 15 wt%, T_c_ and T_m_ appeared at − 108 °C with ΔH_c_ equal to − 48 J/g and − 54 °C with ΔH_m_ equal to 87 J/g, respectively. In solutions with 10 wt% and 15 wt% PS, the ΔH_f,D_ values were 49 J/g and 65 J/g, respectively. Comparing the enthalpy change values between the 15 wt% and 10 wt% PS solutions, at the higher polymer concentration, fewer solvent crystallites were formed in the quenching process due to the influence of the polymer interaction. A similar phenomenon was observed for ΔH_m,D_, i.e., a value of 12 J/g for the 10 wt% PS solution and a value of 21 J/g for the 15 wt% PS solution. In Fig. 2c, when 20 wt% PS was used, T_c_ and T_m_ were − 90 °C and − 57 °C, respectively, with similar absolute values of ΔH_c_ (− 62 J/g) and ΔH_m_ (61 J/g). According to Eq. (1), ΔH_f_ approached 0 J/g, indicating that the solid DMF that was frozen under T_f_ was almost a crystallite-free amorphous phase. The x_c_ generated by cold crystallization at T_c_ can be estimated from ΔH_c_, which was equivalent to the degree of crystallization (x_m_) that disappeared at T_m_. In Fig. 2d, the behavior of the 24 wt% PS solution tended to be the same as that of the 20 wt% solution, except that the absolute values of ΔH_c_ and ΔH_m_ were further reduced.

In Fig. 2e, the 30 wt% PS solution did not show any unique peaks, implying that there was no physical change during the DSC scan. When quenched or heated, the solution contained a sufficient amount of PS compared to the amount of DMF in the solution, which resulted in a sharp increase in viscosity, reduced diffusion, increased physical interactions between molecules, and no crystallite formation. Additionally, no cold crystallization occurred. This phenomenon observed in a 30 wt% PS solution indicates that the state of the polymer and the solvent in the solution was only the amorphous phase without a phase change, even if the cooling and heating processes were repeated, meaning that they were in an amorphous frozen phase at low temperatures and an amorphous liquid phase at high temperatures. The solution in this state must have 100% molecular interaction between the polymer and the solvent, making it similar to the gelation phenomenon. In this regard, comparative experiments were carried out. Typically, gelation experiments are performed by rapidly increasing the viscosity^21^.

**Figure 3:**
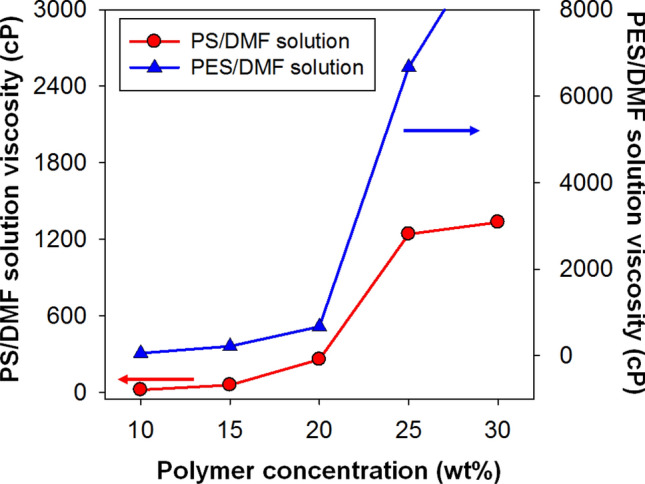
Viscosity variation with the concentration (wt%) of PS/DMF and PES/DMF solutions.

In a simple experiment, as shown in Supplementary Fig. 6, the physical gelation was determined by taking into account the degree of flow when the PS/DMF (30/70) solution was inverted after 24 h of storage at − 5 °C and − 20 °C. In Supplementary Fig. 6, the PS/DMF (30/70) solution flowed well, even when it was inverted. In contrast, the PES/DMF (30/70) solution did not flow. In Supplementary Fig. 1, the ΔH_c_ and ΔH_m_ of the PES/DMF (30/70) solution became 0, and T_c_ and T_m_ disappeared. This phenomenon was the same as that of the PS/DMF (30/70) solution. Figure 3 shows the measurement results by a Brookfield viscometer, revealing a sudden change in viscosity. In both cases, there was a sharp increase in viscosity, but the viscosity of the PS/DMF (30/70) solution was 1,334.5 cP, and the solution could flow. On the other hand, the viscosity of the PES/DMF (30/70) solution was higher than the maximum measurable value of 9,999 cP of the equipment used, and the PES/DMF (30/70) solution did not flow. In this experiment, PS/DMF (30/70) and PES/DMF (30/70) appeared to have lost the T_c_ and T_m_, and the enthalpy changes, ΔH_c_ and ΔH_m,_ were both zero. Thus, it was questionable whether gelation had occurred in the PS/DMF (30/70) solution.

### Enthalpy change behavior of polymer solutions

**Figure 4:**
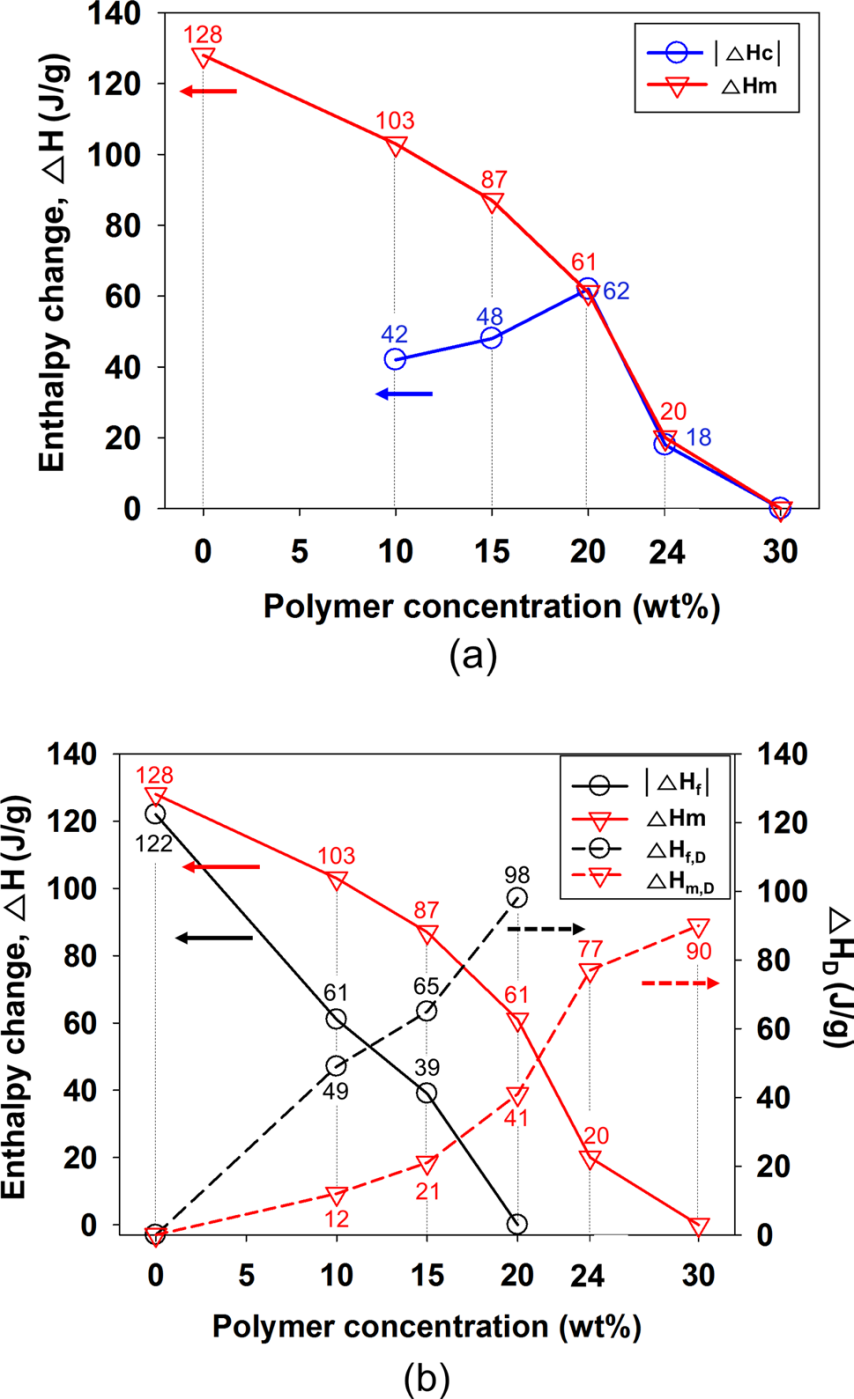
Variation in the phase temperature and enthalpy change with the PS concentration (wt%) of the PS/DMF solutions (refer to Fig. 2): (**a**) for |ΔH_c_| and ΔH_m_ and (**b**) for |ΔH_f_|, ΔH_m_, ΔH_f,D_ and ΔH_m,D_.

Figure 4 shows the enthalpy change trend in Table 1 and Fig. 2.

In Fig. 4a, |ΔH_c_| increased and then began to decrease after reaching ΔH_m,_ which monotonously decreased. In Fig. 4b, |ΔH_f_|, calculated by Eq. (1), decreased monotonically, and ΔH_f,D_ and ΔH_m,D_ increased. The two values reflect the degree of differences between the states in which the polymer and solvent are not supposed to interact with each other and the actual state in which the polymer interacts with the solvent. Thus, the influence between the polymer chains and the solvent molecules could be estimated. For the PS/DMF (20/80) solution, ΔH_f_ was 0, and |ΔH_c_| and ΔH_m_ had the same value. In the PS/DMF (30/70) solution, the interaction between the solvent and polymer was maximized so that all T_c_, T_m_, ΔH_c_ and ΔH_m_ parameters disappeared.

### Schematic diagram of PS/DMF solutions based on DSC measurements

**Figure 5:**
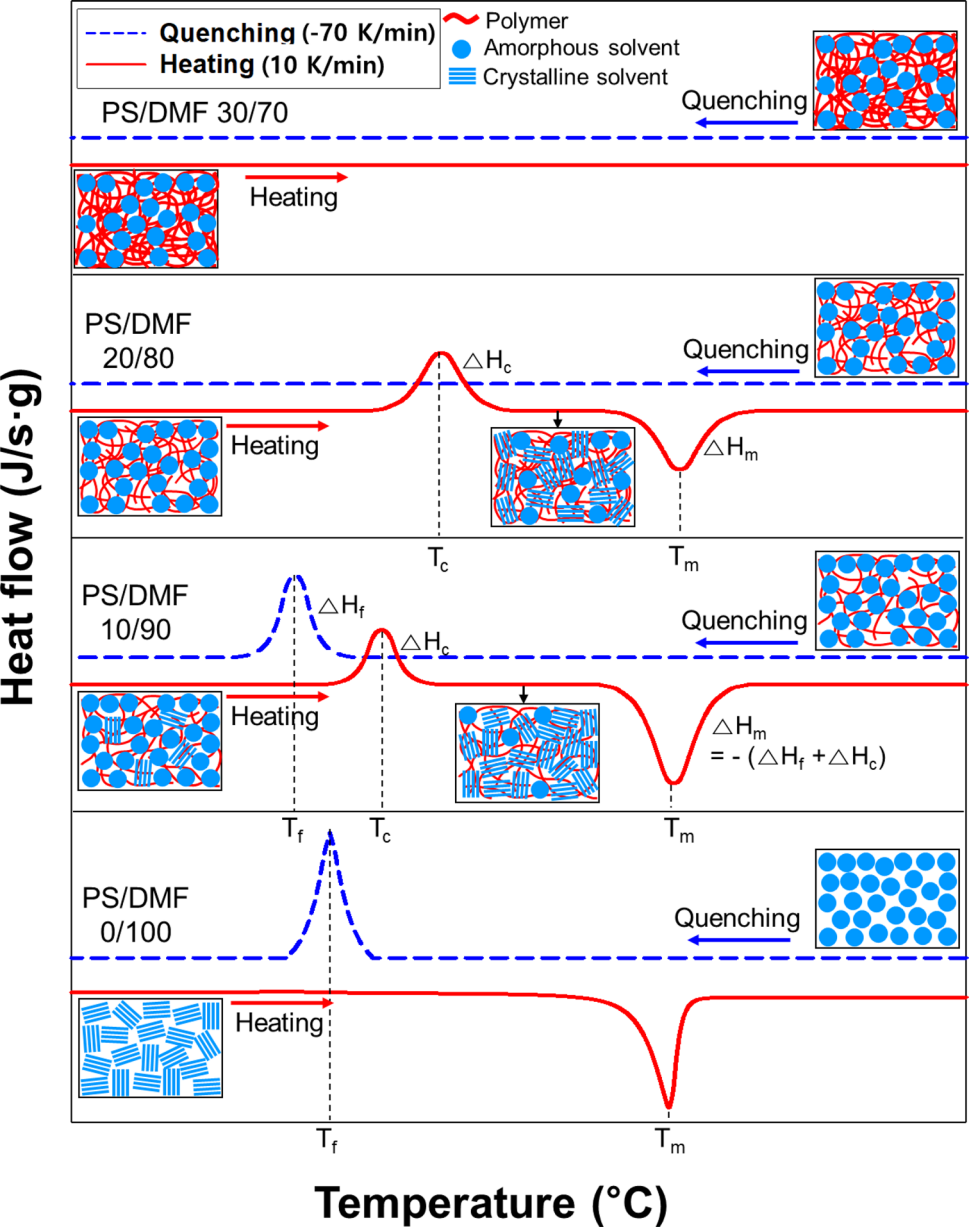
Schematic diagram of the crystalline and amorphous phases of the polymer chains and solvent molecules at different polymer concentrations as a function of temperature. (In the small boxes, each symbol represents the following: polymer, ‘ ’; amorphous solvent molecule, ‘ ’; and solvent crystallite, ‘ ’.)

As described above, Fig. 5 shows the enthalpy change graphically according to the PS concentrations of the crystalline and amorphous phases due to the phase change in the polymer chain and solvent molecules in the PS/DMF solution. The small boxes in Fig. 5 show the phases of the polymer and solvent molecules on the basis of the enthalpy change and degree of crystallization.

In Fig. 5a, the solvent molecules are in the amorphous state at room temperature to temperatures higher than T_m_, and the molecules are represented as ‘ ’. When quenched at − 70 K/min from room temperature to the supercooled region, almost all of the solvent molecules are expected to freeze at T_f_, indicating solvent crystals as ‘ ’. When heated at 10 K/min, it can be seen that the solvent crystal molecules return to an amorphous molecular state ( ) at T_m_, which is supported by the close values of ΔH_f_ and ΔH_m_ of pure DMF listed in Table 1. In a 10 wt% PS solution (Fig. 5b), the polymer chain ( ) and solvent molecules ( ) are homogeneously mixed in the amorphous liquid state at room temperature and distributed as shown in Fig. 5b. When quenched, both frozen amorphous solvent molecules ( ) and crystalline solvent molecules ( ) coexist below T_f_. During the heating scan of the quenched solution, the degree of crystallization (x_c_) of amorphous solvent molecules ( ) increases due to cold crystallization. At T_m_, the solvent molecules ( ,  ) in which the two phases coexist are converted to the liquid amorphous state ( ). In Table 1, the number of solvent crystallites formed at T_c_ in Fig. 5b in the PS/DMF (10/90) solution can be estimated from ΔH_c_ (− 42 J/g), and ΔH_m_ was 103 J/g. According to Eq. (1), ΔH_f_ can be estimated to be approximately − 61 J/g. The molecular arrangement of some amorphous solvent molecules ( ) changed to crystallite solvent molecules ( ) as they passed through T_c_ during the quenching and heating scan cycle, as shown in the small box in Fig. 5b. As shown in Fig. 5c, during the quenching and heating scan of the 20 wt% PS solution, crystallite solvent molecules ( ) were produced at T_c_, and they changed into the amorphous phase ( ) at T_m_. In Table 1, the values of ΔH_c_ and ΔH_m_ were − 62 J/g and 61 J/g, respectively, and their absolute values were almost identical. Based on this result, solvent crystallization did not occur during quenching up to − 170 °C, and no solvent crystallites were formed or existed as a frozen amorphous phase ( ), which would have been generated only by cold crystallization. At room temperature to above T_m_, the solution existed as a liquid amorphous phase ( ), so the molecular arrangements of the PS/DMF (20/80) solution at − 170 °C and room temperature were the same.

In Fig. 5d, the 30 wt% PS solution existed as a homogeneous solution () at room temperature, similar to the 10 wt% and 20 wt% PS solutions, but the enthalpy change (ΔH) due to the phase change during quenching and heating did not appear, and both T_c_ and T_m_ disappeared. In this solution, the phase of the solvent molecules affected by the polymer chain was the same during the following scan cycle, i.e., ① at room temperature, ② quenching below T_f_, ③ heating above T_c_, and ④ returning to room temperature. The results explained that the liquid phases were frozen and then liquefied as an amorphous phase without crystallization. A strong interaction between the polymer and the solvent is necessary to break down the crystallites of the polymer and to dissolve it^36^. On the other hand, the interaction or steric hindrance of the polymer mediates the crystallite formation in the solvent or breaks down the solvent crystallites. As a result, below T_f_, the frozen amorphous phase was completely formed under certain conditions; despite the repeated cooling and heating cycles, crystallization could not occur. Thus, no amorphous phase to crystal phase change could be observed, but a liquid to solid state change could be observed under certain conditions. Both of these phenomena were found to occur simultaneously in a 30 wt% PS solution.

### Applications of enthalpy changes in polymer solutions

#### Pore size distribution of mesoporous materials

**Figure 6:**
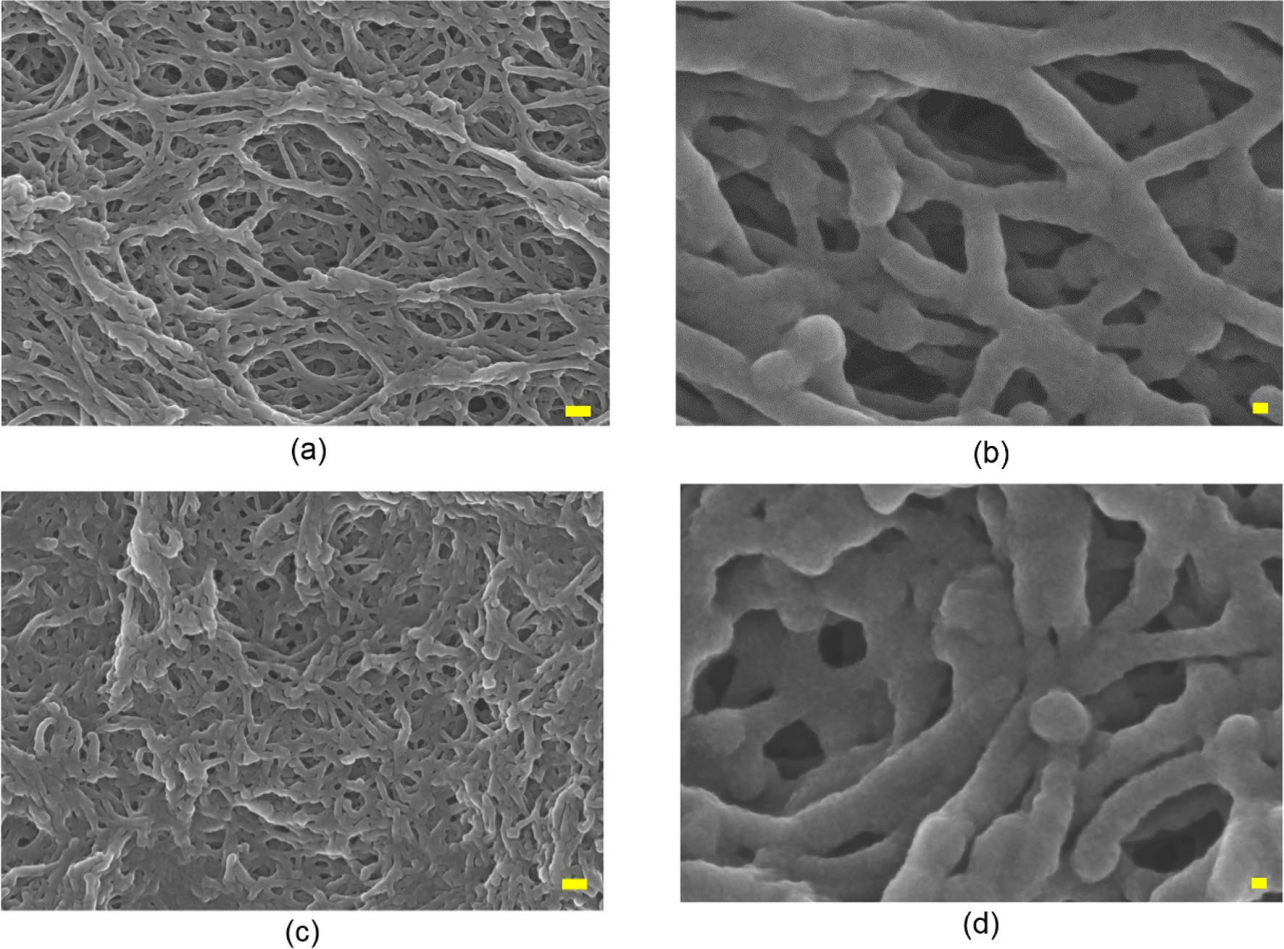
Comparison of the pore characteristics with and without annealing in the preparation of porous membrane materials using the PS/DMF (22/78) solution. (**a**) A cross-sectional SEM photograph of the unannealed porous material (× 50,000 magnification, inserted scale bar; 100 nm) and (**b**) (× 300,000 magnification, inserted scale bar; 10 nm); (**c**) A cross-sectional SEM photograph of the porous material annealed for 24 h (× 50,000 magnification, inserted scale bar; 100 nm) and (**d**) (× 300,000 magnification, inserted scale bar; 10 nm).

In Fig. 6a,b, the cross-sectional scanning electron microscopy (SEM) images of samples prepared with the PS/DMF (22/78) solution without annealing were obtained at magnifications of 50,000 and 300,000, respectively. Figure 6c,d show samples after 24 h of annealing that were maintained at − 80 °C for 24 h. In Supplementary Fig. 5, the average pore sizes measured by a surface area analyzer (Micromeritics, ASAP2420) were 16 nm and 20 nm, and the average pore size increased due to the growth of solvent DMF crystallites during the annealing treatment. The strut structures of PS open cells showed that the strut structures of the samples formed after 24 h of annealing were thicker than those without annealing. In Supplementary Fig. 5, the Brunauer–Emmett–Teller (BET) surface area was measured and found to be 281 m^2^/g and 236 m^2^/g, respectively. Thus, the annealed sample accelerated nucleation and growth of solvent crystallites, resulting in smaller surface areas due to larger open cell pore sizes and thicker struts compared to samples with no annealing, and mesoporous materials with narrower pore size distributions were obtained, as shown in Fig. 6 and Supplementary Fig. 5.

#### A new approach for gelation

Figure 5 shows the crystallization of the solvent molecules and the change in the amorphous phase. The presence of the frozen amorphous phase of solvent molecules was determined by the effect of the polymer concentration on mediating solvent crystallization. In other words, the interaction between solvent and polymer was measured, which might explain the gelling phenomenon. For example, neither the PS/DMF (30/70) solution nor the PES/DMF (30/70) solution showed T_c_ and T_m_, and consequently, the enthalpy changes were all zero. It was concluded that both solutions were gelled.

By applying another method to verify the gelation, in Supplementary Fig. 6, unlike the gelled PES/DMF (30/70) solutions, flow was observed for PS/DMF (30/70) solutions. Then, conditions were applied to prevent gelation of the PS/DMF (30/70) solution, in contrast to the ΔHm test method. Thus, the phenomena in which ΔH_c_, ΔH_m_ and ΔH_f_ are all zero might be proposed as an alternative and new approach for gelation.

## Discussion

The phase change temperature and enthalpy change behavior of polymer solutions in the ultralow temperature region were studied. As the cooling scan rate was increased, the T_f_ of pure DMF changed to − 83 °C, − 96 °C and − 113 °C, but T_m_ was approximately − 61 °C with a ΔH_m_ of 128 J/g, which are close to the literature values. The phase change temperature of the solvent DMF with the cooling and heating scan cycles was confirmed. The PS/DMF solution was quantitatively analyzed for T_c_, T_m_, ΔH_c_ and ΔH_m_ by repeating the cooling and heating processes. When the PS concentrations increased to 10 wt%, 15 wt%, 20 wt% and 24 wt%, T_c_ shifted to − 116 °C, − 108 °C, − 90 °C, and − 75 °C, respectively, and the ΔH_c_ values were − 42 J/g, − 48 J/g, − 62 J/g, and − 18 J/g, respectively, with their absolute values increasing and decreasing; ΔH_m_ simply decreased to 103 J/g, 87 J/g, 61 J/g, and 20 J/g, respectively. Especially in the PS/DMF (30/70) solution, T_c_, T_m_, ΔH_c_ and ΔH_m_ disappeared. The increase in the ΔH_m,D_ value with increasing polymer concentration was in good agreement with the fact that increasing the molecular interactions or affinity between polymer and solvent mediates the formation of solvent crystallites. As the polymer concentration increased, |ΔH_c_| ≈ ΔH_m_ and ΔH_f_ ≈ 0 for PS/DMF (20/80). For PS/DMF (30/70), T_c_ did not appear, T_m_ did not appear, and ΔH_c_ = ΔH_m_ = 0. By adjusting the polymer concentration, the pore size could be controlled. That is, after annealing at T_c_, the average pore size was changed from 16 to 20 nm through the quenching and heating process of the concentrated polymer solution.

We found that the PS/DMF (30/70) solution showed a rapid increase in viscosity due to the strong interaction between the solvent and polymer; however, flow was observed, and the enthalpy change approached zero.

## Methods

### Preparation of the polymer solution

PS (M_n_ = 240,000 g/mol) was supplied by Kumho Petrochemical Ltd. and PES (M_n_ = 75,000 g/mol) was obtained from BASF Ultrason 6020P. DMF (99.0%) and NMP (99.5%) as solvents were purchased from Samchun Chemical Co. PS and PES were dried at 50 °C and 100 °C for 24 h in a vacuum oven to remove moisture completely. Casting solutions were prepared by dissolving PS and PES in DMF and NMP, respectively, at the desired concentration for 3 h at room temperature and stirring at 100 °C for 12 h. Finally, the solution was kept at room temperature for 24 h to remove air bubbles.

### DSC measurements

The quenching and heating data of the polymer solution were measured by a DSC instrument (NETSCH, Polyma 214) equipped with a liquid nitrogen cooler. Aluminum pans were used and tested in a closed state. A DSC sample mass of approximately 10–20 mg was transferred using a micropipette. For the DSC measurements, as shown in Scheme 1, Figs. 1 and 2, the circled numbers ①, ②, and ③ represent the sequence of processes. Specifically, the solution was first cooled to − 170 °C by ① quenching (− 70 K/min) and stabilized for 5 min, heated to room temperature by ② heating (10 K/min), followed by ③ quenching (− 70 K/min) again to − 170 °C. To eliminate the thermal history of the solution, ① quenching was ignored, and the ② heating and ③ quenching results were used, as shown in Figs. 1 and 2.

### Preparation of porous materials

Porous material preparation experiments were carried out in a glove box in a controlled manner under a nitrogen atmosphere. The prepared polymer solutions were dropped on polyethylene terephthalate (PET) films and cast using a casting knife. The cast solution was quenched with liquid nitrogen, and the quenched casting solution was subjected to solvent exchange using nonsolvent methanol in a cryogenic freezer. Porous materials were obtained by drying at 50 °C using a vacuum oven to remove the residual solvent completely.

### SEM observations

The morphology of porous materials from the polymer solution (Supplementary Fig. 5) was visualized with a Hitachi FE-SEM SU8220 Field-Emission Scanning Electron Microscope at the Western Seoul Center, Korea Basic Science Institute. The film sample was completely dried in a vacuum oven at 50 °C and cut using liquid nitrogen. To observe the cross section of the sample, the sample was adhered to the SEM sample holder using carbon adhesive tape and then sputtered at 30-s intervals using an osmium coater (vacuum device, HPC-30). Cross sections of the coated samples were observed at magnifications of 50,000 and 300,000.

### Gas adsorption measurements

The pore size distribution was measured using a volumetric gas adsorption apparatus (Micromeritics, ASAP2420) to confirm the pore size control of the porous materials using a polymer solution (Fig. 6 and Supplementary Fig. 6). One hundred milligrams of the dried sample was filled in a glass sample tube and pretreated at 50 °C to remove the adsorbed substances on the sample for 24 h. Nitrogen adsorption isotherms were measured at 77 K, and the pore distribution was determined using the BET method.

## Supplementary information

Supplementary information.
