# Supplementary material for: Novel behavior in a polymer solution: the disappearance of the melting temperature (Tm) and enthalpy change (ΔHm) of the solvent
Source: Sci Rep. 2020 Aug 7;10:13348. doi: 10.1038/s41598-020-70331-4 (PMC7415149; doi:10.1038/s41598-020-70331-4)
Supplement: Supplementary file 1 — Supplementary information. [file 41598_2020_70331_MOESM1_ESM.pdf]

Supplementary Information

Novel behavior in a polymer solution: the disappearance of the melting temperature (T_m) and enthalpy change ($\triangle H_m$) of the solvent

Mi Rae Kim[1], Hee Jung Park[2], Kang Ho Cheon[1], Choong Kyun Yeom[3] & Kee Yoon Lee[1,*]

[1]Department of Polymer Science and Engineering, Chungnam National University, 99 Daehak-Ro, Yuseong-Gu, Deajeon 34134, Republic of Korea.

[2]Western Seoul Center, Korea Basic Science Institute, 150 Bugahyeon-Ro, Seodaemun-Gu, Seoul, 03759, Republic of Korea.

[3]SepraTek, 730 Gyejok-Ro, Daedeok-Gu, Daejeon, 34396, Republic of Korea.

Correspondence and requests for materials should be addressed to K.Y.L. (email: kylee@cnu.ac.kr)

Dynamic DSC experiments were carried out as shown in Scheme 1 for PES/DMF solutions with PES concentrations of 10, 15, 20 and 30 wt%, respectively.

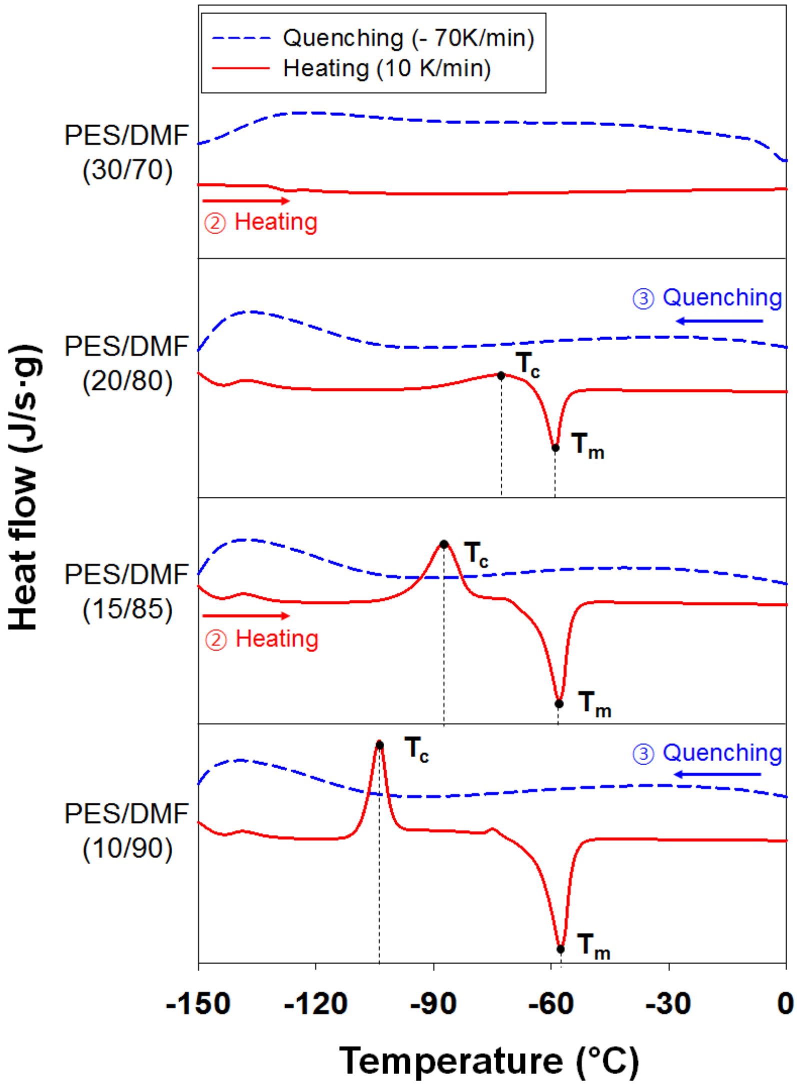

Supplementary Figure 1. Dynamic DSC graph measured for PES/DMF solutions with PES concentrations of 10, 15, 20 and 30 wt%, respectively (refer Scheme 1)

The T_c, T_m, ΔH_c and ΔH_m shown in Supplementary Fig. 1 are summarized in the Supplementary Table 1 below.

Supplementary Table 1. Variation of phase temperature and enthalpy change with PES concentration for PES/DMF solutions (refer to Supplementary Fig. 1).

	T_c (°C)	T_m (°C)	$\triangle H_c$ (J/g)	$\triangle H_m$ (J/g)
PES/DMF(10/90)	-103	-57	-61.7	92.8
PES/DMF(15/85)	-87	-58	-74.1	85.5
PES/DMF(20/80)	-73	-59	-33.4	30.3
PES/DMF(30/70)	-	-	-	-

In PES/DMF solution, the T_c increased with increasing polymer concentration and $\triangle H_m$ decreased. The absolute values of $\triangle H_c$ were similar to those of $\triangle H_m$ in PES/DMF (20/80) solution, however, PES/DMF (30/70) solution did not show any phase change as it was in PS/DMF (30/70).

By changing the solvent from DMF to NMP, thermal experiments were carried out for PS/NMP and PES/NMP solutions. Supplementary Figure 2 shows the graphs obtained from the dynamic DSC experiment of NMP with cooling and heating scan cycle shown in Scheme 1.

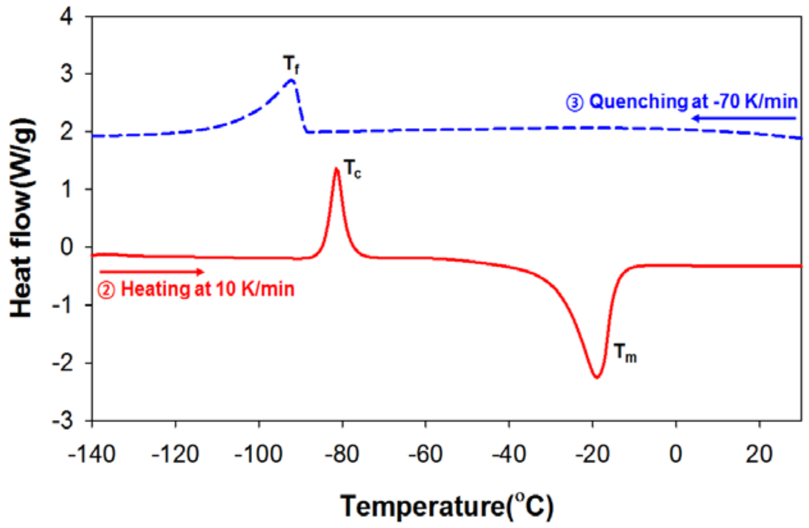

Supplementary Figure 2. Dynamic DSC graph of solvent NMP (refer Scheme 1 for details of scan cycles).

Supplementary Table 2. Phase change temperature and enthalpy change values of NMP solvent (refer Supplementary Fig. 2)

	T_f (°C)	T_c (°C)	T_m (°C)	△H_f (J/g)	△H_c (J/g)	△H_m (J/g)
NMP	-92	-81	-18	39.6	38.3	114.6

The pure NMP solvent showed cold crystallization phenomenon unlike DMF, and T_c appeared at -81 °C. In the polymer/NMP solution, it was difficult to observe the cold crystallization being caused by the polymer, since NMP itself showed the cold crystallization phenomenon.

Dynamic DSC experiments were carried out according to the Scheme 1 as a function of PS concentration of 10, 20, 25 and 30 wt% in PS/NMP solution (Supplementary Fig. 3).

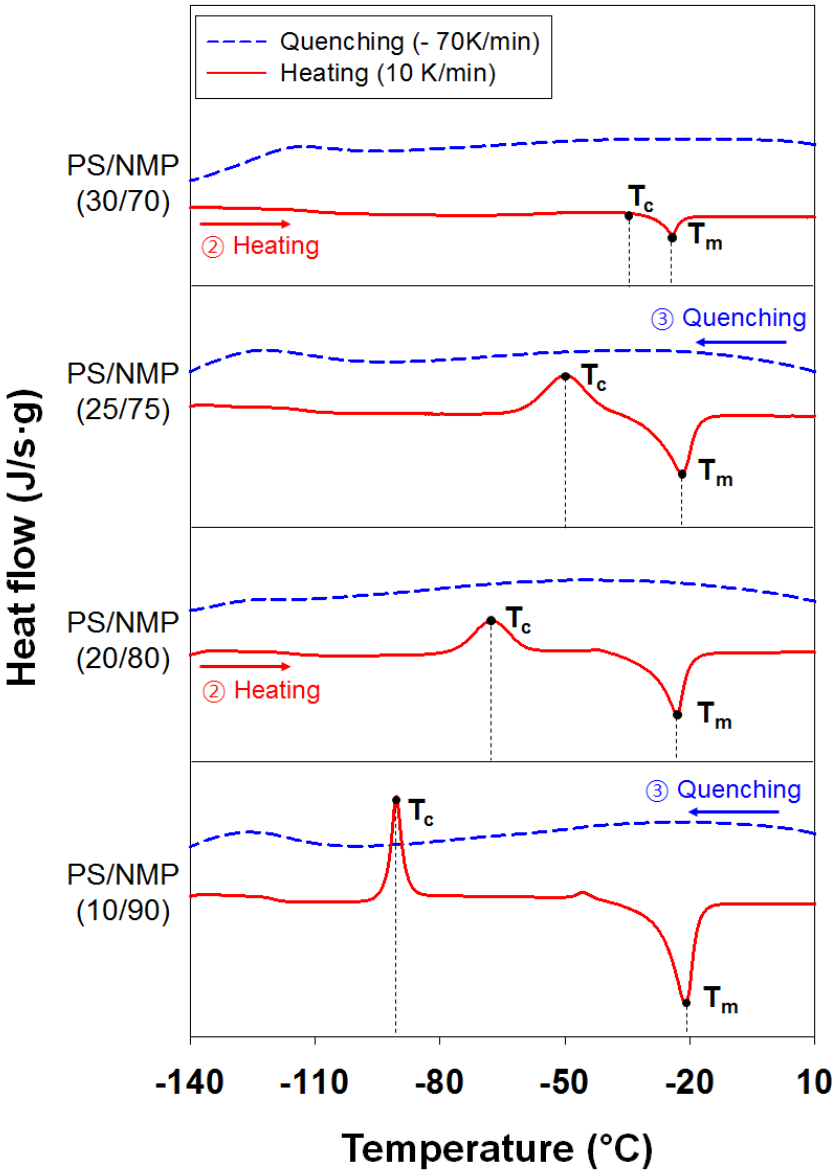

Supplementary Figure 3. Dynamic DSC graphs measured as a function of PS concentration of 10, 20, 25 and 30 wt%, respectively for PS/NMP solutions (refer Scheme 1).

In the PS/NMP (30/70) solution, the T_m was seen and the $\triangle H_m$ appeared as a small peak.

Supplementary Table 3. Phase temperature and enthalpy change of PS/NMP solutions (refer to Supplementary Fig. 3).

	T_c (°C)	T_m (°C)	$\triangle H_c$ (J/g)	$\triangle H_m$ (J/g)
PS/NMP(10/90)	-90	-20	-45.4	86.0
PS/NMP(20/80)	-67	-23	-41.3	55.0
PS/NMP(25/75)	-50	-22	-56.2	62.4
PS/NMP(30/70)	-34	-25	-9.9	9.4

Dynamic DSC experiments were carried out according to Scheme 1, for PES/NMP solution as a function of PES concentration of 10, 15, 20 and 30 wt%, respectively.

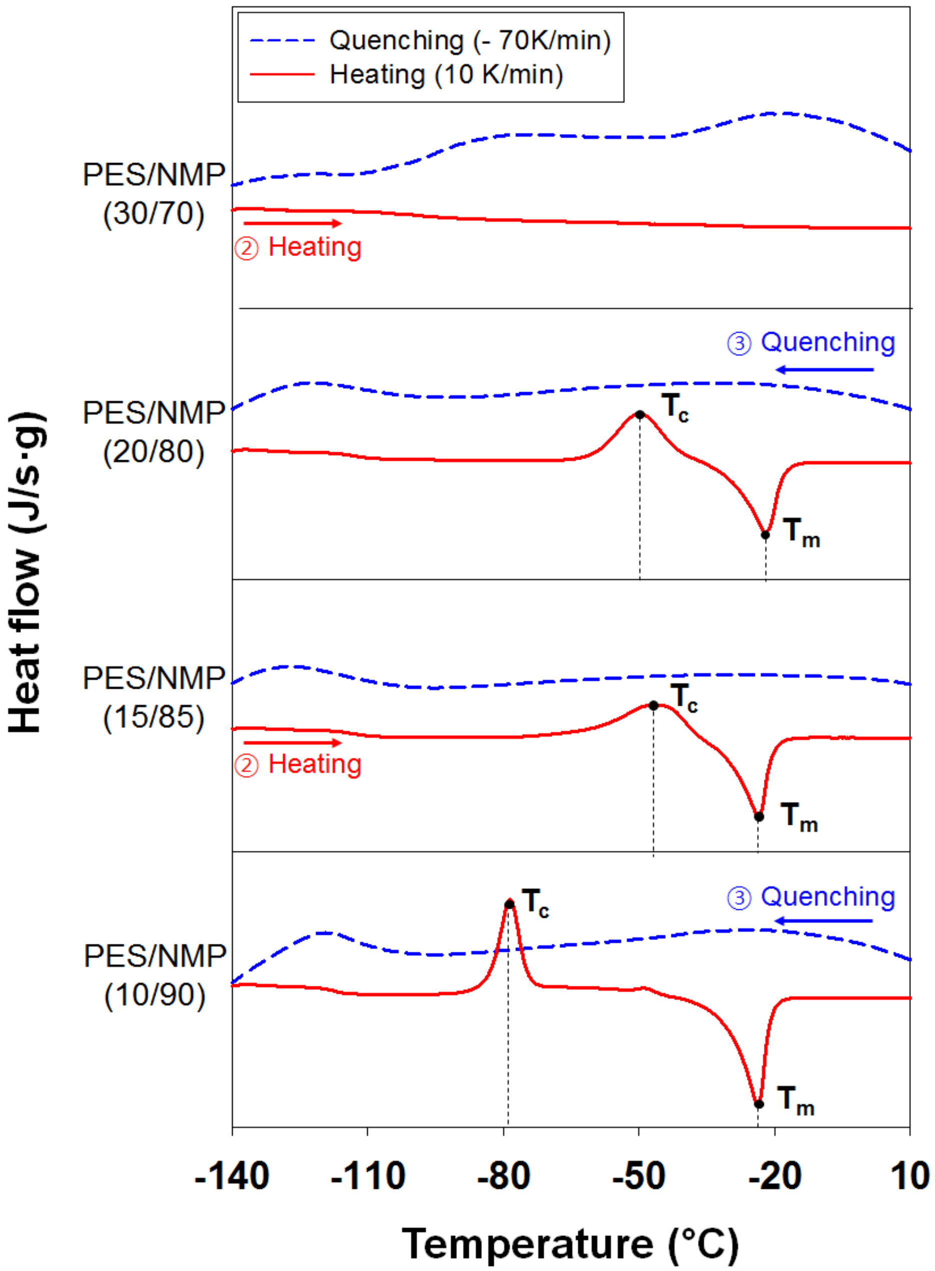

Supplementary Figure 4. Dynamic DSC graphs measured as a function of PES concentration of 10, 15, 20 and 30 wt%, respectively for PES/NMP solutions (refer Scheme 1 for scan conditions).

Both the T_m and the ΔH_m disappeared in PES/NMP (30/70) solution.

Supplementary Table 4. Phase change temperature and enthalpy change values of PES/NMP solutions (refer to Supplementary Fig. 4).

	T_c (°C)	T_m (°C)	$\triangle H_c$ (J/g)	$\triangle H_m$ (J/g)
PES/NMP(10/90)	-78	-23	-53.1	78.7
PES/NMP(15/85)	-46	-23	-57.1	58.3
PES/NMP(20/80)	-35	-25	-59.9	59.3
PES/NMP(30/70)	-	-	-	-

We have carried out the experiment to control the pore size of porous polymer materials using the cold crystallization phenomena. Two experiments were carried out after PS/DMF (22/78) solution was quenched by using liquid nitrogen. In one experiment, the residual solvent DMF was removed by -80 °C methanol without annealing process to make the sample. In another experiment, a mesoporous polymer membrane sample was prepared by removing solvent DMF using methanol, a poor solvent, at -80 °C, after annealing at -80 °C for 24 h. That is, when the solution of the quenched amorphous frozen phases was heated, and reached to T_c, crystallites were generated during annealing for 24 h. The physical properties of the sample without annealing at Tc and the sample with annealing for 24 h were compared and are shown in the following Supplementary Fig. 5.

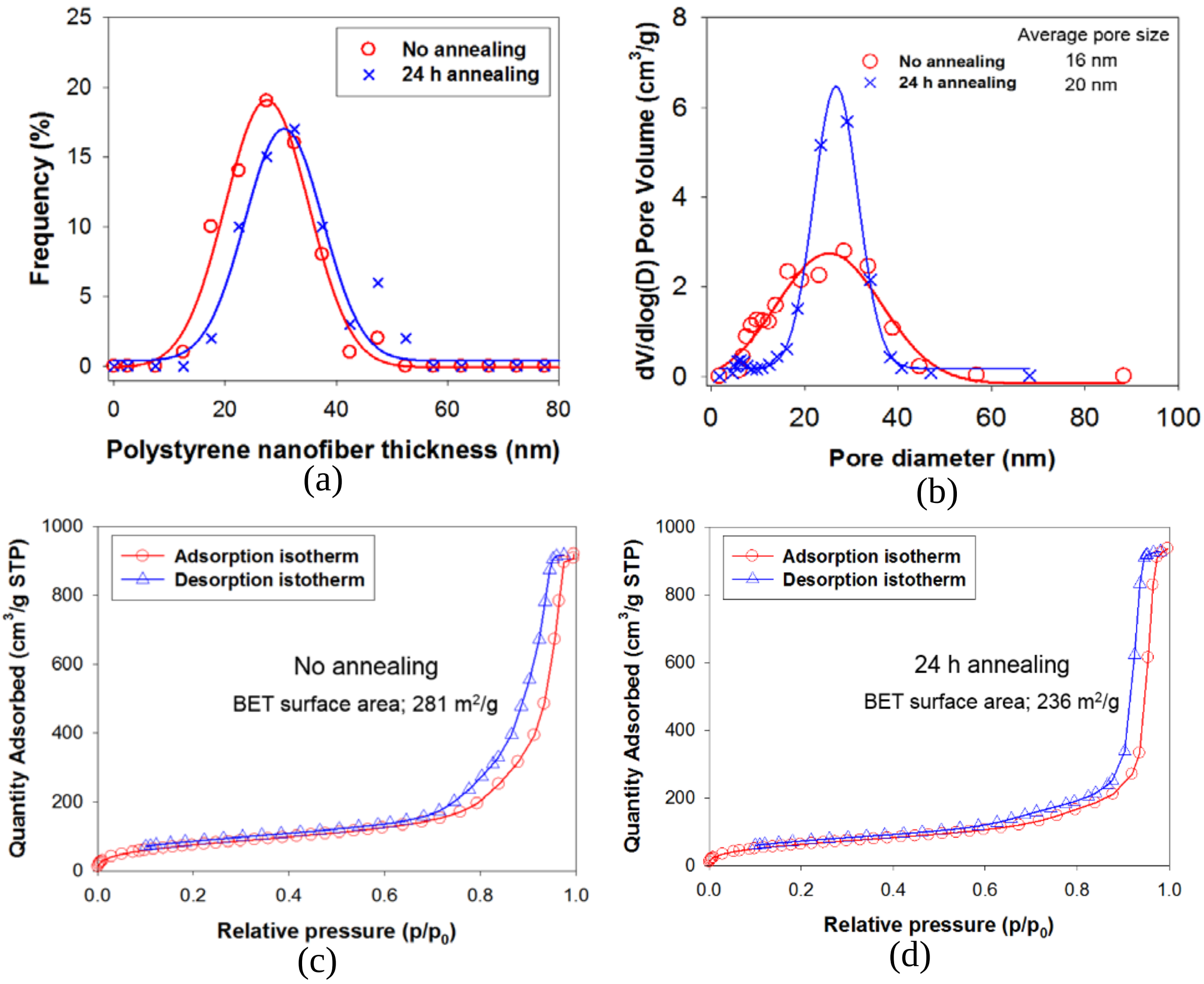

Supplementary Figure 5. Comparison of the pore characteristics with and without annealing in the preparation of porous membrane materials using the PS/DMF (22/78) solution. (a) Comparison of the thickness distribution of the polystyrene nanofiber with and without annealing (refer to Figs. 6 (b) and (d)), (b) Comparison of the pore size distribution of the porous material with and without annealing, (c) Nitrogen adsorption isotherm of the unannealed porous material, (d) Nitrogen adsorption isotherm of the porous material annealed for 24 h.

Supplementary Fig. 5 shows the result of pore characteristics and gas adsorption measurements for PS/DMF (22/78) solution with and without annealing. Supplementary Fig. 5 (a) and (b) show that the thickness was larger and the average pore size distribution was more uniformly narrower distribution for the annealed sample. That is, concerning the nanofiber thickness

shown in Supplementary Fig. 5 (a), the annealed sample showed a thicker nanofibers with an average of 33 nm compared to the average 28 nm of the unannealed sample (ref. Fig. 6 (b) and (d) for SEM images). It was obvious that the solvent molecules formed a larger crystalline phase for the annealed sample and accordingly made the polymer chain thicker. Supplementary Fig. 5 (b) compares the average pore sizes between 16 nm and 20 nm, showing larger and uniform pore size for the annealed sample. Nitrogen adsorption isotherm corresponded to isotherm IV based on the IUPAC, indicating that it was the mesopore pore. BET surface areas were measured at 281 m^2/g and 236 m^2/g, respectively. The BET surface area value was smaller as the pore size was increased by annealing.

The following Supplementary Fig. 6 shows the test results for determining whether the PS/DMF solution and the PES/DMF solution were gelled at -5 °C and -20 °C.

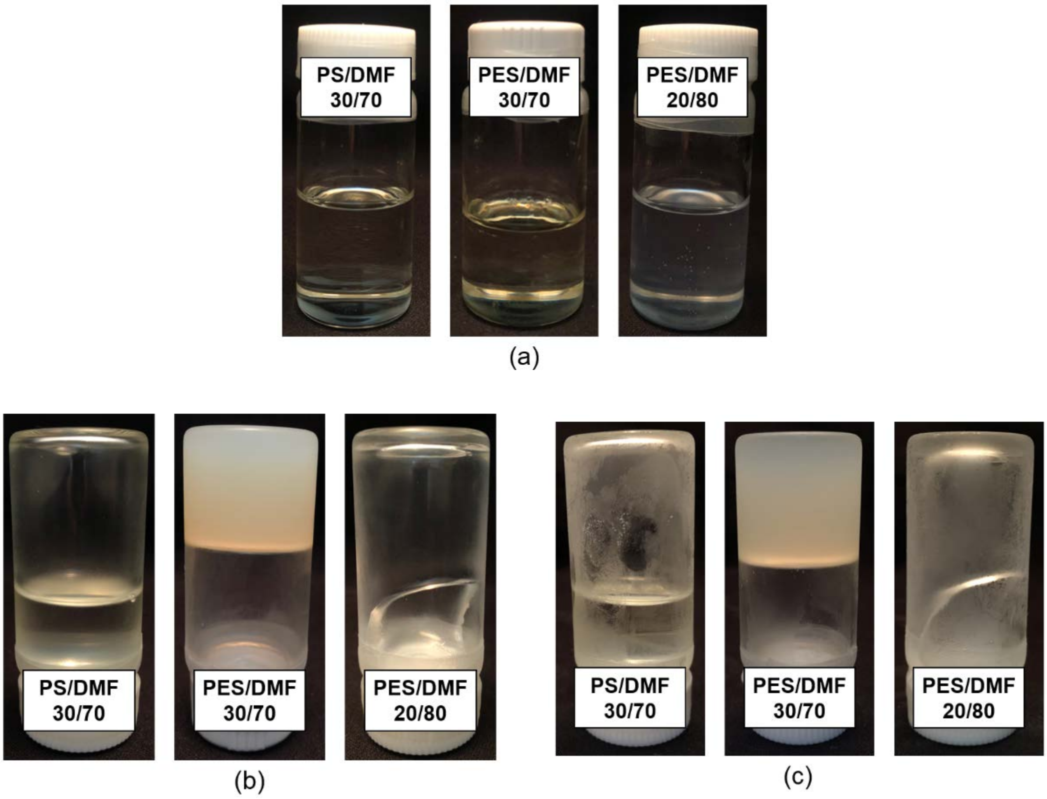

Supplementary Figure 6. Gelation tests for polymer solutions PS/DMF (30/70), PES/DMF (30/70), and PES/DMF (20/80). (a) Polymer solutions at room temperature. (b) After 24 h at -5 °C, the vial was turned upside down. (c) After 24 h at -20 °C, the vial was turned upside down.

Supplementary Figure 6 showed the results of the gel tests for the polymer solutions. The PS/DMF (30/70) can be seen flowing down immediately when the vial was turned upside down at -5 °C and –20 °C after 24 h. PES/DMF (30/70) did not flow down at each temperature, and for PES/DMF (20/80), it flowed down as it was in PS/DMF (30/70).

Our gelation test was based on the fact that there was no ΔH in the case of PS/DMF (30/70) solution in Fig. 2. Considering the results of the physical gelation test conducted at low temperature in Supplementary Fig. 6 and the ΔH measurement result by DSC, we would like to present a basis as a new approach for gelation at polymer/solvent concentrations where △H becomes zero.

Fig. 3 and Supplementary Table 5 show the results of viscosity measurements as the polymer concentrations in PS/DMF and PES/DMF solutions were increased to 10, 15, 20, 25 and 30 wt% respectively.

Supplementary Table 5. Viscosity variation with the concentration (wt%) of PS/DMF and PES/DMF solutions (refer Supplementary Fig. 7).

Polymer concentration (wt%) / Solution	10	15	20	25	30
PS/DMF solution viscosity (cP)	21	59.2	259.1	1241.8	1334.5
PES/DMF solution viscosity (cP)	56.8	219.1	673	6669	∞

The viscosity of the polymer solution was measured by a Brookfield viscometer (DV-1 Prime, Brookfield). Water jacket was used to maintain isothermal conditions for viscosity measurement and kept at 30 °C. The Brookfield viscometer used spindle 18, and 6.7 mL of polymer solution was added to the sample chamber. The the torque value within 100 or less was measured with a shear rate of 0.5 to 100 rpm. The average value of the viscosity measured at each shear rate was obtained and the viscosity values are shown in Fig. 3 and Supplementary Table 5.

In both solutions of PS/DMF and PES/DMF, the viscosities increased as the polymer concentrations increased. In particular, the PES/DMF (30/70) solution gelled, therefore, the viscosity was rendered too high to measure, because of the measurable limit value of 9999cP for the equipment in this laboratory. The following Supplementary Fig. 8 showed the results from the dynamic DSC experiment of PS/DMF (10/90) solution. The experiment was repeated several times to confirm the reproducibility of the experimental values (refer Scheme 1 dynamic quenching and heating scan process).

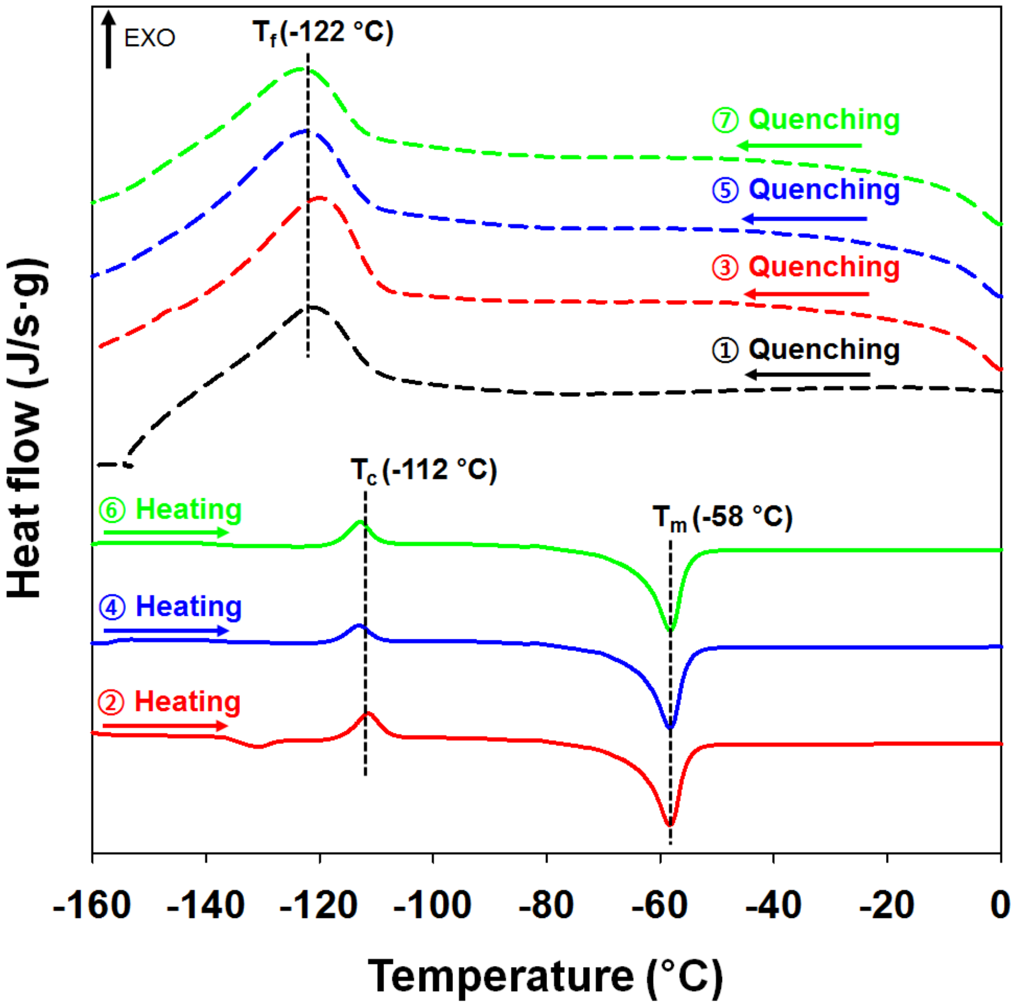

Supplementary Figure 7. Dynamic DSC graphs with repeated scan cycles for PS/DMF (10/90) solution (refer Scheme 1).

Each phase change temperature of T_c, T_m and T_f was measured to give consistently the same value during the repeated scan cycles. This proved obviously that the DSC experiment data were reproducible.
